# Prevalence of Common Child Mental Health Disorders Using Administrative Health Data and Parent Report in a Prospective Community-Based Cohort from Alberta, Canada: Prévalence des troubles communs de santé mentale de l’enfant à l’aide des données de santé administratives et des rapports des parents dans une cohorte prospective communautaire d’Alberta, Canada

**DOI:** 10.1177/07067437241271708

**Published:** 2024-08-21

**Authors:** N. Racine, T. Pitt, S. Premji, S.W. McDonald, S.B. Patten, S. Tough, S. Madigan

**Affiliations:** 1 School of Psychology, 151181 University of Ottawa, Ottawa, Ontario, Canada; 2 27338 Children’s Hospital of Eastern Ontario Research Institute, Ottawa, Ontario, Canada; 3 Department of Paediatrics, 2129 University of Calgary, Calgary, Alberta, Canada; 4 Provincial Population and Public Health, 3146 Alberta Health Services, Calgary, Alberta, Canada; 5 Centre for Health Economics, 8748 University of York, Heslington, York, UK; 6 Department of Community Health Sciences, 2129 University of Calgary, Calgary, Alberta, Canada; 7 Department of Psychiatry, 103136 Peter Lougheed Hospital, Calgary, Alberta, Canada; 8 Department of Psychology, 2129 University of Calgary, Calgary, Alberta, Canada; 9 157744 Alberta Children's Hospital Research Institute, Calgary, Alberta, Canada

**Keywords:** ADHD, mood disorder, anxiety disorder, prevalence, administrative data, early identification, TDAH, troubles de dépression, troubles de l'anxiété, prévalence, données administratives, identification précoce

## Abstract

**Objective:**

Knowing the prevalence of mental health difficulties in young children is critical for early identification and intervention. In the current study, we examine the agreement among three different data sources estimating the prevalence of diagnoses for attention deficit hyperactivity disorder (ADHD) and emotional disorders (i.e., anxiety or mood disorder) for children between birth and 9 years of age.

**Methods:**

Data from a prospective pregnancy cohort was linked with provincial administrative health data for children in Alberta, Canada. We report the positive agreement, negative agreement, and Cohen's Kappa of parent-reported child diagnoses provided by a health professional (“parent report”), exceeding a clinical cut-off on a standardized questionnaire completed by parents (the Behavior Assessment System for Children, 3rd edition [“BASC-3”]), and cumulative inpatient, outpatient, or physician claims diagnoses (“administrative data”).

**Results:**

Positive and negative agreement for administrative data and parent-reported ADHD diagnoses were 70.8% and 95.6%, respectively, and 30.5% and 94.9% for administrative data and the BASC-3, respectively. For emotional disorders, administrative data and parent-reported diagnoses had a positive agreement of 35.7% and negative agreement of 96.30%. Positive and negative agreement for emotional disorders using administrative data and the BASC-3 were 20.0% and 87.4%, respectively. Kappa coefficients were generally low, indicating poor chance-corrected agreement between these data sources.

**Conclusions:**

The data sources highlighted in this study provide disparate agreement for the prevalence of ADHD and emotional disorder diagnoses in young children. Low Kappa coefficients suggest that parent-reported diagnoses, clinically elevated symptoms using a standardized questionnaire, and diagnoses from administrative data serve different purposes and provide discrete estimates of mental health difficulties in early childhood.

**Plain Language Title:**

Prevalence of child mental health disorders according to different data sources in Canada

## Plain Language Summary

Knowing the prevalence of mental health difficulties in young children is critical for informing mental health policy and decision-making. Yet, different sources yield different estimates and we do not know how these estimates compare. In the current study, we examine the agreement among three different information sources estimating the prevalence of diagnoses for attention deficit hyperactivity disorder (ADHD) and emotional disorders (i.e., anxiety or mood disorder) for children between birth and 9 years of age. To estimate the prevalence of mental disorders, we asked parents if their child had ever been diagnosed, we asked parents to complete a questionnaire using clinical symptom cut-offs for diagnosis, and we looked at data collected in the health care system to see if a child was ever diagnosed by a healthcare provider. We found that for ADHD, parent report that their child had received a diagnosis and their child having received a diagnosis in the healthcare system were similar. There were larger differences between a parent report of elevated symptoms on a questionnaire and whether they had been diagnosed by a healthcare provider. For emotion disorders, there were larger differences between parent report that their child had received a diagnosis and whether one was documented in the health record. Overall, there was somewhat low agreement between these three sources of data. We conclude that the different sources of data used in this study provide different estimates of ADHD and emotional disorder diagnoses in children. Therefore, when trying to understand the burden of child mental health disorders in young children, it is important to consider multiple sources to obtain a comprehensive picture of the issue.

## Résumé

**Objectif:**

Connaître la prévalence des problèmes de santé mentale chez les jeunes enfants est essentiel pour une identification et une intervention précoces. Dans la présente étude, nous examinons la concordance entre trois sources de données différentes estimant la prévalence des diagnostics de trouble déficitaire de l'attention avec hyperactivité (TDAH) et de troubles émotionnels (c'est-à-dire anxiété ou trouble de l'humeur) chez les enfants entre la naissance et 9 ans.

**Méthode:**

Les données ont été liées aux données de santé administratives provinciales pour enfants en Alberta, Canada. Nous signalons l’entente positive, l’entente négative et le Kappa de Cohen des diagnostics d’enfant fournis par un professionnel de la santé (« rapport de parent »), le dépassement d'un seuil clinique sur un questionnaire standardisé rempli par les parents (le Behaviour Assessment System for Children, 3e édition (« BASC-3 »)) et les diagnostics cumulatifs de patients hospitalisés, ambulatoires ou de réclamations médicales (« données administratives »).

**Résultats:**

La concordance positive et négative pour les données administratives et les diagnostics de TDAH signalés par les parents était de 70,8% et 95,6%, respectivement, et de 30,5% et 94,9% pour les données administratives et le BASC-3, respectivement. Les troubles émotionnels, les données administratives et les diagnostics déclarés par les parents présentaient une concordance positive de 35,7% et une concordance négative de 95,0%. La concordance positive et négative pour les troubles émotionnels utilisant les données administratives et le BASC-3 était respectivement de 20,0% et 87,4%. Les coefficients Kappa étaient généralement faibles, ce qui indique une mauvaise concordance corrigée du hasard entre ces sources de données.

**Conclusions:**

Les sources de données mises en évidence dans cette étude fournissent un accord disparate sur la prévalence des diagnostics de TDAH et de troubles émotionnels chez les jeunes enfants. Les faibles coefficients Kappa suggèrent que les diagnostics rapportés par les parents, les symptômes cliniquement élevés à l'aide d'un questionnaire standardisé et les diagnostics tirés de données administratives servent des objectifs différents et fournissent des estimations discrètes des difficultés de santé mentale dans la petite enfance.

## Introduction

In Canada, approximately 1 in 5 children under the age of 17 years meet the criteria for a mental health disorder and less than one-third receive the mental health care they need.^1^ Mental illness in children and youth costs the Canadian economy more than $4 billion annually, and these estimates are only for emotional disorders such as anxiety and depression.^2^ Consequently, prevention is critical. A particularly important time to monitor, assess, and treat mental health needs is during early childhood. Research has shown that one-quarter of mental disorders have their onset when children are younger than 14 years,^3^ with even earlier onset for anxiety and behaviour disorders^4^ and ADHD.^5^ Furthermore, many mental health challenges identified in early childhood persist 1–3 years later.^6^ These findings illustrate the importance of monitoring the prevalence of mental disorders in children when opportunities for early intervention have the potential for the greatest impact.^7,8^

There are many information sources that can be used to assess the prevalence of early childhood mental health disorders. Identifying the prevalence of mental health disorders involves a categorical classification (present or absent) of whether an individual has symptoms that would meet the criteria for the diagnosis of a mental health disorder. One potential information source is to examine the number of children who exceed a clinical cut-off using a standardized questionnaire, which is often associated with a diagnosis of a disorder. Structured interviews conducted by healthcare professionals with parents/caregivers of young children that include a series of binary questions to arrive at a diagnosis are also frequently used to identify mental health disorders in children and youth.^9^ Prevalence can also be assessed through parent reports of child diagnoses received in public or non-public health settings.^10^

Another method to estimate prevalence is using administrative health records, which involves the collection of medical and community drug prescription data as part of routine record-keeping within the public health sector to identify the number of children diagnosed with mental disorders.^11,12^ Within Canada, administrative data provide comprehensive coverage of community physician, inpatient, and outpatient services, and are linkable via a unique patient identifier.^13^ Although several studies have validated the use of administrative data relative to other diagnostic methods for mental health disorders in adults^14,15^ very few studies have been conducted in children. Indeed, the investigation of mental disorders in young children under 10 years of age remains a neglected area of research, despite the potential for investments at these early ages to mitigate poor outcomes.^16^ No research to date, to our knowledge, has uniquely focused on the prevalence of mental disorders in children under the age of 9 years, when early identification of bourgeoning mental health concerns may be most salient.

One study investigated the concordance of mental health diagnoses between administrative data and general population survey data in Ontario, Canada for children between 4 and 17 years of age.^17^ Findings indicated that administrative and survey data had low sensitivity and high specificity for diagnoses, whereby the apparent prevalence of mental health diagnoses was significantly higher using population survey data than administrative data.^17,18^ These findings suggest that the prevalence of mental health disorders may be dependent on the information source. In the absence of a gold standard, examining positive and negative agreement among information sources can provide insight into the extent to which there is overlap in these approaches and whether administrative health data provides a useful understanding of mental health disorder diagnoses in young children.

### Research Objective and Questions

The objective of the current study was to examine the agreement in the prevalence of common mental health disorders (i.e., ADHD and emotional disorders) in children between birth and 9 years of age using parent-reported diagnoses provided by a health professional (“parent report”), exceeding a clinical cut-off using a standardized questionnaire (“BASC-3”), and cumulative inpatient, outpatient, or physician claims diagnoses (“administrative data”). Specific research questions were as follows: (a) What is the prevalence of ADHD and emotional disorder diagnoses in children under the age of 9 years across three information sources (i.e., administrative data, parent-reported diagnoses, and exceeding a clinical cut-off on a questionnaire)?; (b) What is the positive and negative agreement between administrative data and parent report of ADHD and emotional disorder diagnoses? (c) What is the positive and negative agreement between administrative data and exceeding the clinical cut-off on a parent-reported questionnaire for ADHD and emotional disorder diagnoses? Based on previous research,^17^ we hypothesize that the prevalence of ADHD and emotional disorders will differ across information sources.

## Method

Two data sources were used for the current study: questionnaires completed by mothers who participated in the prospective All Our Families Cohort (AOF) study and Alberta-based inpatient, outpatient, and physician claims administrative data. Using the AOF study, two estimates of mental health diagnoses for children were used: parent-reported diagnoses that their child had received and exceeded a clinical cut-off on a standardized mental health symptom questionnaire. These sources were linked using unique maternal personal health numbers (PHN). Ethics approval was received from the Conjoint Health Research Ethics Board, University of Calgary (Ethics ID 130868). A waiver of consent was provided to access child administrative health records. Permission to access vital statistics (births) and practitioner claims data was provided by Alberta Health.

### Sample

The AOF study (*n* = 3387) is a prospective community-based pregnancy cohort based in Calgary, Alberta, Canada. The cohort was designed to examine risk and protective factors for early child development and family well-being.^19^ Details on recruitment, eligibility, data collection, and measurement have been described elsewhere.^20^ Participants in The All Our Families cohort are representative of the demographics of those living in Calgary, Alberta, and Canada. Women in the AOF cohort generally had higher annual incomes and were more likely to be married than those in Alberta and Canada.^20^

### Data Linkage

**Figure 1.**
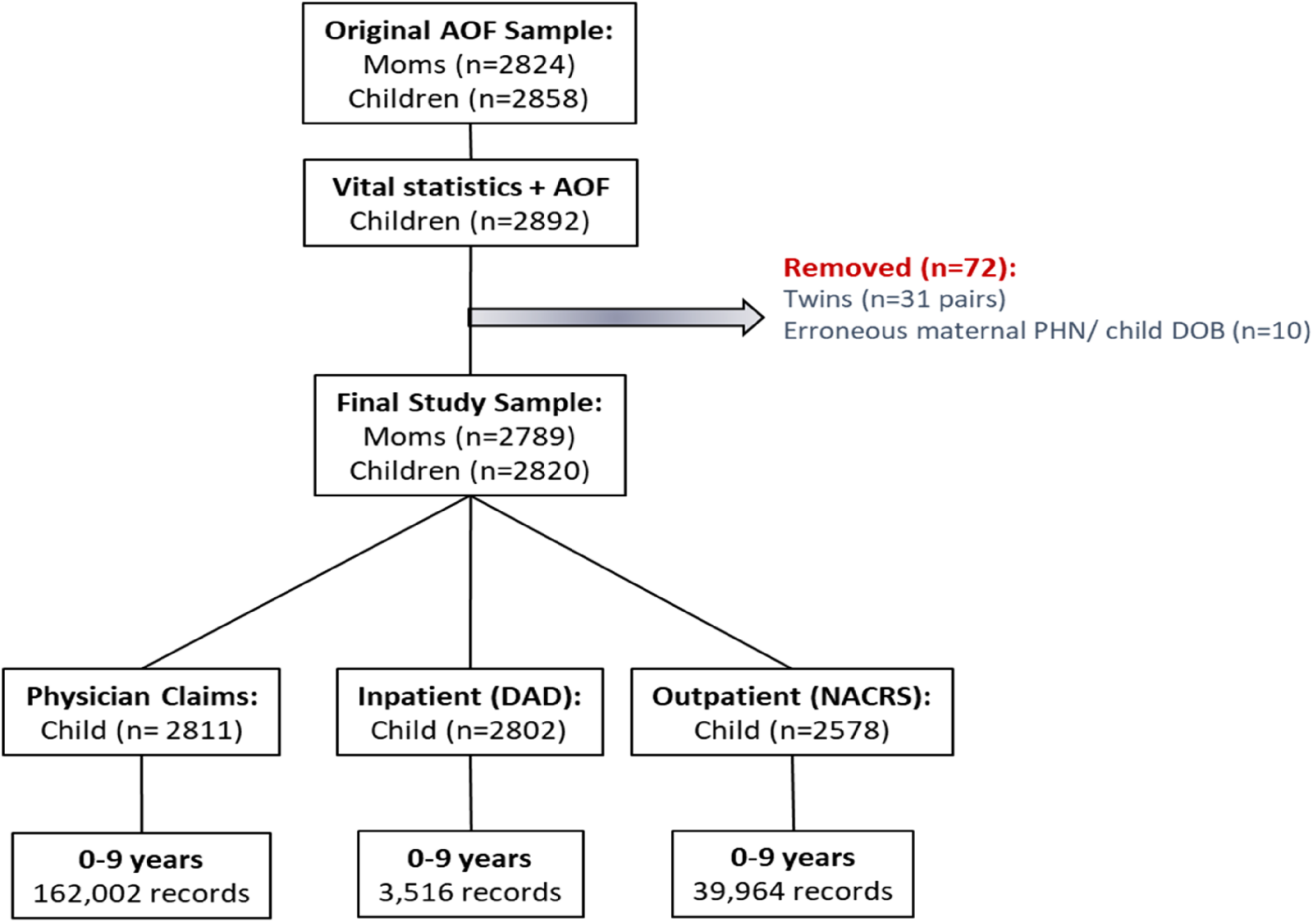
Administrative health data linkage.

At recruitment, mothers in AOF were asked for consent to link to their medical records (*n* = 2824 consented). Using maternal PHN and child date of birth, child PHNs were obtained using Alberta vital statistics (birth) records, and linked to inpatient (Discharge Abstract Data, or DAD), outpatient (National Ambulatory Care Reporting System, or NACRS), and practitioner claims data to support the identification of mental health diagnoses for children between birth and 9 years of age. These data sets require diagnostic information to be entered in the International Classification of Disease (ICD) 9 or 10 format. Administrative data provides information on many different types of disorders including oppositional disorders, developmental disorders, and autism spectrum disorders. For the purposes of the current study, we focus on ADHD and emotional disorders as they are the most common in young children. Following cohort linkage and cleaning, the eligible sample for this study consisted of 2820 children who engaged in over 20,000 health service contacts between birth and 9 years of age (Figure 1). Of these, 2814 children (99.8%) had at least one health service contact beyond their delivery record within the Alberta public health sector and were included in the final sample for this study.

### Measures

#### Exceeding the Clinical Cut-Off On a Standardized Questionnaire

In the 5- and 8-year surveys, mothers completed the Behavior Assessment System for Children (BASC-3) standardized questionnaire.^21^ The BASC-3 is a widely used tool to assess mental health difficulties and has strong psychometric properties.^21^ Standardized T-scores for combined sex were calculated, with higher scores indicating greater symptoms. For ADHD, children were identified as being likely to have a diagnosis if they were reported to have a T-score ≥ 70 on either the inattention or hyperactivity subscales.^21^ For emotional disorders, children were identified as likely to have a diagnosis if they were reported as having a T-score ≥ 70 on either the anxiety or depression subscales.^21^ A T-score greater than 70 indicates that symptoms are at the 98th percentile or higher. Children were classified as having been diagnosed with the disorder using this method if the clinical cut-off was exceeded at either 5 or 8 years of age. Previous research has demonstrated that for ADHD, 77% of ADHD cases can be positively diagnosed using the BASC^22^ and that BASC can adequately differentiate between children with and without ADHD.^23^ Previous research has found that mean scores on the attention problems and hyperactivity subscales for children diagnosed with ADHD can be lower than 70, indicating that using the cut-off of 70 in the current study is a conservative approach.^24^ For emotional disorders, evidence of sensitivity and specificity for the BASC-3 are more limited, however, other versions of the BASC are associated with other well-established measures of child mental health symptoms have been established.^25^

#### Parent-Reported Mental Health Diagnoses

In the 5-year AOF survey, mothers were asked to report whether their child had *experienced the following health conditions* before age 5 for the following: (a) ADHD and (b) a mental health problem. Mothers specified what mental health problem(s) the child experienced in an open-text question and only depression and anxiety (e.g., generalized anxiety disorder, social anxiety, phobia) were included in this study. In the 8-year AOF survey, mothers were asked whether their child had been diagnosed by a health practitioner since Grade 1 (5 or 6 years of age) for the following: (a) ADHD and (b) a mental health problem (e.g., anxiety or depression). If yes for a mental health problem, the parent specified and only those pertaining to anxiety and depression were retained. The 5- and 8-year parent reports were combined, and a new variable was generated to indicate if the child had been diagnosed (yes = 1, no = 0) with ADHD and/or an emotional disorder at either time point. Parent report of a child diagnosis has been shown to be a valid method for obtaining information about a child's diagnostic status in children with autism spectrum disorder.^26^

#### Administrative Data

Child ADHD diagnoses comprise ICD-9 and ICD-10 codes as identified through two previous studies.^12,17^ An existing case definition adopted by the Canadian Institutes of Health Information (CIHI) for the surveillance of child mental health disorders was used to identify the relevant ICD-9 and ICD-10 diagnostic codes for child emotional disorders, which comprised mood and anxiety disorders.^27^ Supplementary Table 1 provides the case definitions adopted for this study. Similar to previous studies linking the AOF survey to parent mental health diagnoses in administrative data,^28,29^ ADHD, anxiety, and mood disorder diagnoses were defined using the 1 hospitalization (inpatient or outpatient), 2 physician claims (1H2P) method over a 12-month period, which is a validated algorithm for depression and ADHD in Canada.^12,14^ We calculated the lifetime prevalence of receiving an ADHD diagnosis and of receiving an anxiety or mood disorder diagnosis before age 9 years.

### Statistical Analyses

For objective 1, we estimated the prevalence of mental health disorders across information sources. For objectives 2 and 3, we examined the positive and negative agreement as well as Cohen's Kappa (κ) with 95% confidence intervals (CI) between the BASC-3, parent report, and administrative data. Like previous research,^17^ we did not apply a gold standard, but rather examined agreement among sources. Positive agreement represents the likelihood of a child being diagnosed with ADHD or an emotional disorder information across both information sources, while negative agreement represents the likelihood of a child not being diagnosed with ADHD or an emotional disorder across both information sources under consideration.

### Missing Data

Of the 2814 children in our linked sample, approximately 55% completed the parent report and BASC-3 questions during the 5 and/or 8-year AOF data collection period. The majority of missing data was attributed to longitudinal participant attrition (unit non-response) in the AOF cohort,^20^ a common disadvantage of community-based cohort studies.^30^ Bivariate analyses examining the association between missing data and various family socio-demographic characteristics are presented in Supplementary Table 2. Additionally, having missing data in the AOF survey was not related to being diagnosed in administrative health data (see Supplementary Table 3). While methods to address missing data, such as multiple imputation, have been gaining momentum in recent years as a potential solution to support medical research,^31^ for this study, we proceeded with a complete case analysis to ensure we were accurately capturing estimates of agreement and κ between information sources.

## Results

Socio-demographic characteristics are presented in Table 1. Table 2 shows the estimates of ADHD and emotional disorders (mood and anxiety) for children aged 9 years. Positive agreement, negative agreement, and κ between information sources are presented in Table 3. Comparing administrative data and parent report for ADHD yielded a positive agreement of 70.8%, a negative agreement of 95.6%, and κ = 0.496 (95% CI: 0.404–0.588). For administrative data and the BASC-3 cut-offs, positive agreement was 30.5%, negative agreement was 94.9%, and κ = 0.198 (95% CI: 0.106–0.289). For emotional disorders, administrative data and parent report yielded a positive agreement of 35.7%, negative agreement of 96.3%, and κ = 0.118 (95% CI: 0.016–0.221), whereas positive agreement was 20.0%, negative agreement was 87.4%, and κ = 0.011 (95% CI: −0.019–0.040) between administrative data and the BASC-3 questionnaire. Finally, comparing parent report to elevated symptoms in the BASC-3 yielded a positive agreement of 34.8%, negative agreement of 96.0%, and κ = 0.313 (95% CI: 0.220–0.406) for ADHD, and a positive agreement of 9.6%, negative agreement of 97.8%, and κ = 0.110 (95% CI: 0.050–0.170) for emotional disorder.

**Table 1.** Sample Characteristics.

Characteristic (*n* = 2,814)	*N* (%)
*Child* s*ex*	
*Male*	1,489 (53.0)
*Female*	1,323 (47.0)
*Maternal age at delivery*	
* < 35 years*	2,196 (80.2)
≥ *35 years*	543 (19.8)
*Parity*	
*No previous baby*	1,371 (49.3)
*Previous baby*	1,410 (50.7)
*Household family income*	
*<$80,000*	808 (29.8)
≥$*80,000*	1,904 (70.2)
*Ethnicity*	
*White*	2,214 (79.2)
*Non-White*	583 (20.8)
*Maternal education*	
*High* s*chool or* l*ess*	293 (10.5)
*More than high school*	2,505 (89.5)
*Marital status*	
*Married or* c*ommon* l*aw*	2,660 (95.1)
*Single*	138 (4.9)
*Maternal mental health history*	
*No*	1,879 (67.1)
*Yes*	920 (32.9)

**Table 2.** Prevalence of ADHD and Emotional Disorders up to 9 Years of Age.

	Administrative data (*n* = 1,556)*	Parent report (emotional disorder *n* = 1550; ADHD *n* = 1,540)	BASC-3 (*n* = 1,556)	Any data source (*n* = 1,556)*	Diagnosis in all data sources (*n* = 1,556)*
	*N* (%)				
ADHD	59 (3.8)	111 (7.2)	94 (6.1)	168 (10.8)	12 (0.8)
Emotional disorder	15 (1.0)	62 (4.0)	197 (12.7)	235 (15.1)	1 (0.1)
Mood disorder	9 (0.6)	5 (0.3)	111 (7.1)	121 (7.8)	0 (0.0)
Anxiety disorder	6 (0.4)	60 (4.0)	138 (8.9)	170 (10.9)	1 (0.1)
Any disorder (ADHD or emotional)	69 (4.4)	125 (8.0)	243 (15.6)	327 (21.0)	23 (1.5)

* Children for whom there was incomplete AOF survey data were excluded.

**Table 3.** Agreement Across Different Data Sources.

	Positive agreement	Negative agreement	Kappa (95% CI)
	Administrative data and parent report		
ADHD	70.8%	95.6%	0.496 (0.404–0.588)
Emotional disorder	35.7%	96.3%	0.118 (0.016–0.221)
ADHD or emotional disorder	60.9%	94.4%	0.399 (0.309–0.488)
	Administrative data and BASC-3		
ADHD	30.5%	94.9%	0.198 (0.106–0.289)
Emotional disorder	20.0%	87.4%	0.011 (-0.019–0.040)
ADHD or emotional disorder	46.4%	85.8%	0.146 (0.087–0.205)
	Parent report and BASC-3		
ADHD	34.8%	96.0%	0.313 (0.220–0.406)
Emotional disorder	9.6%	97.8%	0.110 (0.050–0.170)
ADHD or emotional disorder	24.3%	95.0%	0.240 (0.175–0.305)

## Discussion

**Figure 2.**
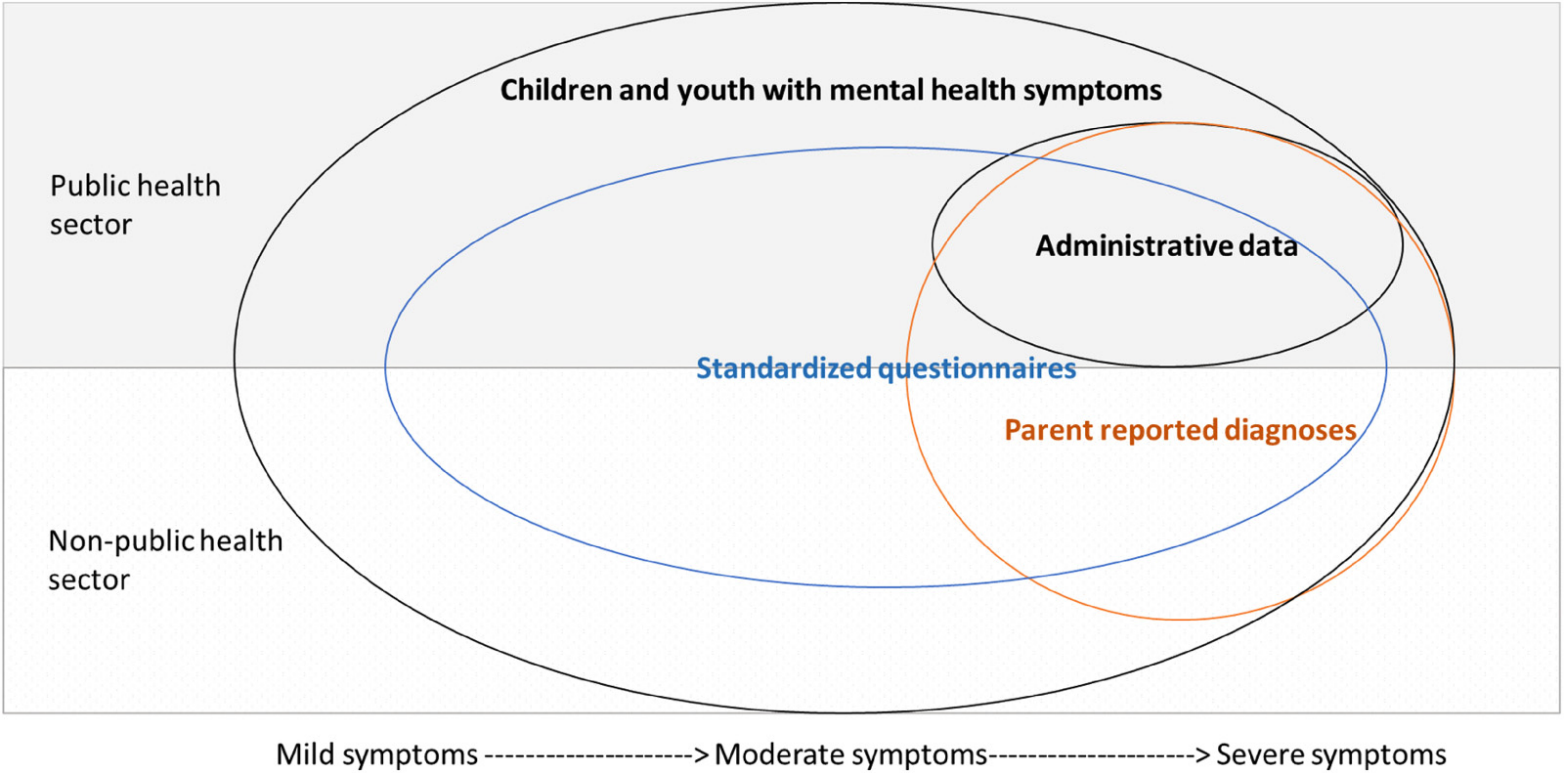
Hypothetical representation of how different data sources can capture unique segments of the population.

Growing evidence suggests that mental health difficulties among children and youth are on the rise^32^ and more needs to be done to support identification before adolescence, when opportunities to intervene have the greatest potential to benefit both children and society.^7,8^ Data are also needed to accurately estimate the prevalence to help inform whether there is sufficient capacity within the public health sector to support and treat mental health disorders in children and youth and monitor changes in their mental health over time. Using linked data from the AOF prospective community-based cohort and administrative data, this study aimed to examine agreement for diagnosis with a child mental health disorder using three distinct information sources: exceeding a clinical cut-off using a standardized questionnaire, parent-reported diagnoses provided by a health professional, and inpatient, outpatient, or physician claim diagnoses using ICD-9 and ICD-10 codes in children before age 9 years. Understanding how many children may require support related to an emotional disorder or ADHD within the mental health system can be useful for resource planning. Study findings suggest that estimates varied significantly by information source, with the highest estimates being from exceeding cut-offs on standardized questionnaires and the lowest estimates being found in administrative data (Table 2). These findings strengthen recent findings,^11^ which also suggest that varying information sources capture slightly different yet overlapping groups of individuals. We provide a theoretical representation of how these information sources may capture different individuals in the population (see Figure 2).

Using criteria outlined by Landis and Koch to interpret agreement,^33^ results indicate that negative agreement across information sources ranged from 85.8% to 97.8%, whereas positive agreement ranged from 9.6% to 70.8% (Table 3). Agreements were generally moderate at best. It should be noted that Kappa values are impacted by low prevalence rates, which is why we also consider the positive and negative agreements.^34^

The highest positive agreement was between administrative data and parent report of an ADHD diagnosis by a healthcare professional (70.8%, κ = 0.496). Notably, when comparing administrative data with exceeding the clinical cut-off on a questionnaire for emotional disorders, the overall κ was poor (κ = 0.011), and 95% CI ranged from −0.019 to 0.040, indicating agreement may be worse than chance.^34^ Others have observed a similar discordance when examining the agreement of mental health diagnoses between survey and administrative data in youth.^17^

Interestingly, there was greater agreement amongst measures of ADHD than emotional disorders. Diagnoses of ADHD are typically assigned by a medical professional, as there is an associated pharmacological intervention, which is likely why ADHD diagnoses are well documented in administrative data. Parents may also more readily notice externalizing symptoms, like those present for ADHD, and may therefore be more apt to bring these to the attention of their healthcare provider.

In contrast, emotional disorders, including anxiety or depression, may be more likely diagnosed by community psychologists or school-based services and are therefore less likely to be recorded in routine administrative health data. Those relying on administrative information sources to estimate the prevalence of emotional disorder are likely underestimating this disorder. This finding also aligns with the broader literature in this area showing that 62% of parents report receiving mental health support outside the health system, more specifically with school-based services.^1^

More generally, the low κ estimated in Table 3 suggests that these measurement strategies should not be regarded as interchangeable. As presented as a theoretical depiction in Figure 2, each measurement strategy likely captures different concepts, where exceeding a clinical cut-off on the BASC-3 questionnaire appears to identify the largest group of children with mild, moderate, or severe symptoms. On the other hand, parent-reported diagnoses indicate the frequency of diagnoses obtained, which provides information on cases being detected from either the public or non-public health sectors. Finally, administrative data estimates provide information for those at the severe end of the spectrum who access the public health sector (e.g., emergency department or inpatient services) for support. Administrative data estimates indicate the prevalence of severe disorders but may also point to potential failures of the public health sector to address these disorders in a timely and effective manner. Each information source therefore serves different purposes and provides distinct estimates of mental health diagnoses in early childhood. These sources may be used individually to inform resource planning. Specifically, it is important to consider these disparate information sources to have a comprehensive understanding of children's mental health presentations to inform policies targeting child mental health. For example, if emotional disorders are not frequently captured in administrative data, surveillance that involves surveys or diagnostic interviews to understand the prevalence of emotional disorders among children may be necessary.

### Strengths and Limitations

First, there is potential for selection bias arising from differences in the characteristics of mothers who provided versus did not provide consent to link to their medical records^29^ (*n* = 2824 and *n* = 563, respectively) and from differences in those who completed the AOF surveys at 5 and 8 years relative to the full sample (55%). This potential for selection bias likely resulted in an underestimate of diagnoses for ADHD and emotional disorders. Further, while the use of the AOF prospective cohort provided a representative urban population of women and their children,^20^ caution is provided in generalizing these findings to a rural demographic. These variations in participant socio-demographics may additionally contribute to differences in the prevalence rates found in our study.^35^ For example, women in the present study were of higher income and higher education, which means they may have had better access to mental health services outside the public health system (e.g., psychologists), which tend to be fee for service. As such, reports of parent-reported diagnoses may have been higher in this sample.

Second, questions with regards to parent-reported diagnoses differed slightly between the 5-year and 8-year time points. Specifically, parents were asked to report on whether their child had experienced a mental health condition at age 5 years versus having been diagnosed by a health practitioner at age 8 years. This difference may have resulted in a slight overestimate of conditions reported by parents at the 5-year time point. Additionally, the validity and reliability of parent-reported ADHD and emotional disorder diagnoses are unknown.

Third, although there is previous evidence in the adult literature for the use of the 1H2P as a validated algorithm for identifying depression and ADHD in administrative data in Canada,^12,14^ this method has not been validated for anxiety. Future research to establish this method with childhood anxiety is needed.

Fourth, the current study relied exclusively on maternal reports. Although parents, especially mothers, are the most frequent reporters in child mental health studies,^6,36^ future research would benefit from the inclusion of multi-informant reporting, including the perspective of fathers. Finally, we also note a potential for misclassification bias arising from data coding errors of a random nature, which likely also resulted in an underestimate of diagnoses. It is also unknown to what extent findings from the current study would generalize to other less common disorders, such as autism spectrum disorder or developmental disorders.

## Conclusion

While assessments of ADHD and emotional disorders in the current study reflect varying strategies that may be used to determine overall prevalence, low Kappa coefficients suggest that parent-reported diagnoses, exceeding a clinical cut-off on a standardized questionnaire, and diagnoses from administrative data serve different purposes and provide discrete estimates of mental health disorders in early childhood. Overall, there was greater concordance among measures of ADHD than emotional disorders, suggesting that administrative data may underestimate the prevalence of emotional disorders. While administrative data identifies individuals who have accessed the health system for their concerns, parent-reported diagnoses may provide a more comprehensive indication of diagnoses received outside the healthcare system. Additionally, the BASC questionnaire is likely over-inclusive of individuals who have elevated symptoms but may not necessarily meet the criteria for a diagnosis. Diagnostic interviews of a large representative sample may provide the most accurate estimate of prevalence. Taken together, these information sources capture different groups of individuals, which may require different levels of support and resource allocation.

## Supplemental Material

sj-docx-1-cpa-10.1177_07067437241271708 - Supplemental material for Prevalence of Common Child Mental Health Disorders Using Administrative Health Data and Parent Report in a Prospective Community-Based Cohort from Alberta, Canada: Prévalence des troubles communs de santé mentale de l’enfant à l’aide des données de santé administratives et des rapports des parents dans une cohorte prospective communautaire d’Alberta, Canada Supplemental material, sj-docx-1-cpa-10.1177_07067437241271708 for Prevalence of Common Child Mental Health Disorders Using Administrative Health Data and Parent Report in a Prospective Community-Based Cohort from Alberta, Canada: Prévalence des troubles communs de santé mentale de l’enfant à l’aide des données de santé administratives et des rapports des parents dans une cohorte prospective communautaire d’Alberta, Canada by N. Racine, T. Pitt, S. Premji, S.W. McDonald, S.B. Patten, S. Tough and S. Madigan in The Canadian Journal of Psychiatry
