# Supplementary material for: Prevalence of Common Child Mental Health Disorders Using Administrative Health Data and Parent Report in a Prospective Community-Based Cohort from Alberta, Canada: Prévalence des troubles communs de santé mentale de l’enfant à l’aide des données de santé administratives et des rapports des parents dans une cohorte prospective communautaire d’Alberta, Canada
Source: Can J Psychiatry. 2024 Aug 21;69(10):768–77. doi: 10.1177/07067437241271708 (PMC11485669; doi:10.1177/07067437241271708)
Supplement: sj-docx-1-cpa-10.1177_07067437241271708 - Supplemental material for Prevalence of Common Child Mental Health Disorders Using Administrative Health Data and Parent Report in a Prospective Community-Based Cohort from Alberta, Canada: Prévalence des troubles communs de santé mentale de l’enfant à l’aid [file sj-docx-1-cpa-10.1177_07067437241271708.docx]

## Supplementary Table 1. Case definitions

|  | **ICD-9 Codes** | **ICD-10 Codes** |
| --- | --- | --- |
| **ADHD** | 314.0, 314.1, 314.2, 314.8, 314.9 | F90, F90.1, F90.2, F90.8, F90.9 |
| **Mood Disorder** | 296.00, 296.01, 296.02, 296.03, 296.04, 296.05, 296.06, 296.40, 296.41, 296.42, 296.43, 296.44, 296.45, 296.46, 296.5, 296.51, 296.52, 296.53, 296.54, 296.55, 296.56, 296.6, 296.61, 296.62, 296.63, 296.64, 296.65, 296.66, 296.7, 296.8, 296.89, 301.13, 296.2, 296.21, 296.22, 296.23, 296.24, 296.25, 296.26, 296.3, 296.31, 296.32, 296.33, 296.34, 296.35, 296.36, 300.4, 311, 293.83, 296.9 | F30, F31, F32, F33, F341, F53, F340, F348, F349, F38, F39, F531 |
| **Anxiety Disorder** | 300.00, 300.01, 300.02, 300.21, 300.22, 300.23, 300.29, 309.21, 313.23, 293.84, 293.89 | F40, F41, F930, F931, F932, F940 |

Supplementary Table 2. Sociodemographic differences for individuals with missing versus complete data.

| **Variable** | **Total** | **Complete** | **Any BASC Q7 or Q8 Missing** | **p-value** |
| --- | --- | --- | --- | --- |
| **Maternal age** | | | | |
| Continuous | 30.70 (4.50) | 31.03 (4.35) | 30.35 (4.63) | **0.0001** |
| **Maternal age** | | | | |
| <35 years | 2194 (80.16) | 1116 (79.04) | 1080 (81.36) | 0.123 |
| ≥35 years | 543 (19.84) | 296 (20.96) | 247 (18.64) |  |
| **Income** | | | | |
| <$80,000 | 808 (29.74) | 363 (26.27) | 445 (33.36) | **<0.001** |
| ≥$80,000 | 1904 (70.26) | 1019 (73.73) | 885 (66.64) |  |
| **Parity** | | | | |
| No Previous Baby | 1370 (49.3) | 720 (50.42) | 651 (48.11) | 0.224 |
| Previous Baby | 1409 (50.7) | 708 (49.58) | 702 (51.89) |  |
| **Ethnicity** | | | | |
| Caucasian | 2212 (79.14) | 1181 (82.24) | 1033 (75.86) | **<0.001** |
| Other | 583 (20.86) | 255 (17.76) | 328 (24.14) |  |
| **Marital Status** | | | | |
| Married/Common Law | 2659 (95.1) | 1387 (96.52) | 1273 (93.6) | **<0.001** |
| Single (including single with partner) | 137 (4.9) | 50 (3.48) | 88 (6.4) |  |
| **Time in Canada** | | | | |
| Born/Lived ≥ 5 years | 2521 (90.55) | 1320 (92.18) | 1203 (88.83) | **0.003** |
| Lived in Canada < 5 years | 263 (9.45) | 112 (7.82) | 151 (11.17) |  |
| **Reported history of depression** | | | | |
| No History | 1879 (67.18) | 982 (68.34) | 897 (65.96) | 0.18 |
| History | 918 (32.82) | 455 (31.66) | 465 (34.04) |  |
| **Child Sex** | | | | |
| Male | 1489 (52.99) | 748 (52.05) | 741 (53.97) | 0.309 |
| Female | 1321 (47.01) | 689 (47.95) | 634 (46.03) |  |
| **Child GA** | | | | |
| <37weeks | 192 (7.29) | 92 (6.55) | 100 (8.12) | 0.122 |
| ≥37 weeks | 2443 (92.71) | 1312 (93.45) | 1132 (91.88) |  |
| **Education** | | | | |
| High School Or Less | 292 (10.44) | 97 (6.76) | 196 (14.32) | **<0.001** |
| Some Or Completed Post Secondary | 2504 (89.56) | 1337 (93.24) | 1168 (85.68) |  |

Note: A cut off of <$80,000 was used to represent approximately one standard deviation below the median household family income level in Calgary, Alberta, which was $105,060 in 2019. This value has been used in several studies previously published from the AOF cohort ^23, 31, 33^.

Supplementary Table 3. Differences in diagnoses in administrative data for those with complete versus missing data in the All Our Families Cohort study.

| Variable | Total | Complete | Missed Either 5-year or 8-year Survey | p-value |
| --- | --- | --- | --- | --- |
| Emotional Disorder (Administrative Health Data) |  |  |  |  |
| No | 2788 | 1541 | 1247 | 0.805 |
| Yes | 26 | 15 | 11 |  |
| ADHD (Administrative Health Data) |  |  |  |  |
| No | 2698 | 1497 | 1201 | 0.327 |
| Yes | 116 | 59 | 57 |  |
